# Editorial: Strategies for crops to confront extreme weather and pests/diseases

**DOI:** 10.3389/fgene.2026.1795055

**Published:** 2026-02-27

**Authors:** Lianjun Wang, Meng Kou, Degao Liu, Shaopei Gao

**Affiliations:** 1 Institute of Food Crops, Hubei Academy of Agricultural Sciences, Wuhan, China; 2 Xuzhou Institute of Agricultural Sciences in Jiangsu Xuhuai District/Key Laboratory of Biology and Genetic Breeding of Sweetpotato, Ministry of Agriculture and Rural Affairs, Xuzhou, China; 3 Department of Plant and Soil Science, Institute of Genomics for Crop Abiotic Stress Tolerance, Texas Tech University, Lubbock, TX, United States; 4 Key Laboratory of Sweetpotato Biology and Biotechnology, Ministry of Agriculture and Rural Affairs, College of Agronomy and Biotechnology, China Agricultural University, Beijing, China

**Keywords:** biotechnology, breeding, climate change, food security, genomics, oat (*Avena sativa*), orphan crops, sweetpotato (*Ipomoea batatas*)

## Introduction

Global agriculture faces mounting threats from climate change and its associated extremes, including droughts, floods, heatwaves, and soil salinization, coupled with increasing pressures from pests and diseases. These challenges compromise crop productivity and undermine food security worldwide (Siddique et al., 2021). The current reliance on a narrow range of staple crops has resulted in genetic uniformity, which limits adaptive potential. Many orphan crops exhibit inherent tolerance to abiotic and biotic stresses and represent a valuable genetic resource for breeding more resilient varieties (Mabhaudhi et al., 2019; Zhang et al., 2018). This editorial highlights how the integration of orphan crops with modern genomic and biotechnological tools can accelerate the development of crops capable of withstanding extreme weather events and pest or disease outbreaks, aligning directly with the research priorities of this Research Topic.

## The vulnerability of modern cropping systems

**FIGURE 1:**
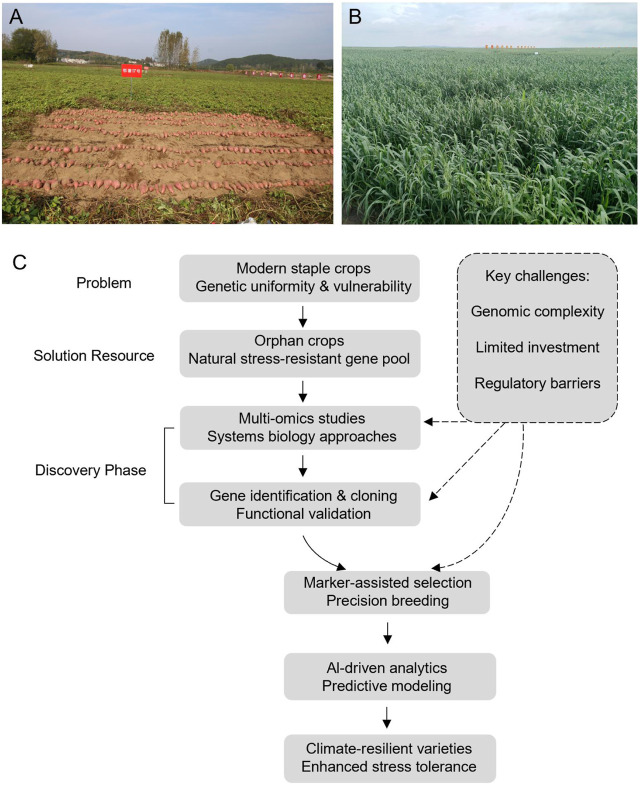
Framework for developing stress-resilient crops using orphan genetic resources. **(A)** Sweetpotato. **(B)** Oat. **(C)** The linear workflow progresses from identifying genetic resources in orphan crops, through multi-omics analysis and gene discovery, to precision breeding enhanced by AI-driven analytics. The output is climate-resilient varieties with enhanced stress tolerance. Key challenges (right) influence the gene discovery and breeding stages.

Despite high productivity, monoculture-based agricultural systems are highly vulnerable to climatic extremes and pathogen outbreaks. Historical instances of crop failure underscore the risks posed by genetic homogeneity. Many orphan crops, such as sweetpotato (*Ipomoea batatas* [L.] Lam.) and oat (*Avena sativa* L.), have evolved under marginal environmental conditions and possess robust resistance mechanisms (Figures 1A,B). Their integration into farming systems can help buffer against yield losses and reduce dependence on chemical inputs. Although recent data indicate increased cultivation of several orphan crops, their potential remains largely underutilized in mainstream agriculture.

## Orphan crops as reservoirs of stress resilience

Orphan crops offer a rich source of genetic traits conferring tolerance to drought, salinity, temperature extremes, and resistance to pests and diseases (Mabhaudhi et al., 2019). For instance:

- Sweetpotato demonstrates high productivity across diverse climates, from tropical to temperate regions.
- Oat thrives in cool, moist environments with modest soil fertility requirements.

Studying these species provides insights into stress-response pathways and facilitates the identification of candidate genes for improving major crops. For example, Nie et al. present a genome-wide analysis of the oat TCP gene family and examine its expression under abiotic stress. Yu et al. functionally characterize the *IbXTH16* gene, demonstrating its role in enhancing cold tolerance in transgenic sweetpotato. Additionally, Wang et al. systematically identify GATA family genes in sweetpotato and analyze their expression responses to abiotic stress.

## Genomic and biotechnological enablers

Advances in genomics, gene editing, and phenomics are revolutionizing the improvement of orphan crops. High-throughput sequencing has uncovered stress-tolerance genes in species such as sweetpotato and oat. CRISPR-Cas9 enables precise editing of domestication and resistance traits, often through targeting orthologues of known genes. Integrated approaches, including *de novo* domestication, speed breeding, and AI-driven phenomics, can significantly shorten breeding cycles and accelerate the development of climate-resilient varieties Figures 1C. These strategies directly support the cloning, validation, and deployment of resistance genes, a core focus of this Research Topic.

## Integration into sustainable and resilient agrosystems

Incorporating orphan crops into crop rotations and intercropping systems enhances biodiversity, improves soil health, and reduces pest pressure. Beyond agronomic benefits, the nutritional richness of many orphan crops addresses dietary deficiencies and meets growing demand in health-conscious markets. Si et al., for instance, systematically evaluate the effects of Chinese herbal medicine extracts on the postharvest storage quality of sweetpotato. Such practices align with the principles of ecological intensification and help reduce the environmental footprint of agriculture.

## Challenges and future directions

Several hurdles remain, including genetic complexity, self-incompatibility, seed shattering, and limited genomic resources. Regulatory barriers and insufficient investment also slow progress. Future efforts should prioritize:

- Gene cloning and functional analysis of stress-tolerance traits,
- Marker-assisted selection and genomic prediction for orphan crops,
- Multi-omics studies to elucidate resistance networks,
- Policy frameworks that promote germplasm exchange and breeding investment.

The integration of AI and big data analytics will further enhance predictive breeding and digital agriculture, ushering in a new era of climate-adaptive crop development.

## Conclusion

Orphan crops, supported by genomic tools and intelligent breeding frameworks, offer a viable pathway toward climate-resilient agriculture. By harnessing their innate stress tolerance and accelerating genetic gains through biotechnology, we can diversify food systems and strengthen global food security. This editorial underscores the timely importance of research on resistance mechanisms and genetic improvement, which is the central theme of this Research Topic, for developing crops capable of thriving in the face of climatic and biotic challenges.
